# Nigrostriatal Dopaminergic Denervation Does Not Promote Impulsive Choice in the Rat: Implication for Impulse Control Disorders in Parkinson’s Disease

**DOI:** 10.3389/fnbeh.2018.00312

**Published:** 2018-12-13

**Authors:** Robin Magnard, Yvan Vachez, Carole Carcenac, Sabrina Boulet, Jean-Luc Houeto, Marc Savasta, David Belin, Sebastien Carnicella

**Affiliations:** ^1^INSERM U1216 and Univ. Grenoble Alpes, Grenoble Institut des Neurosciences (GIN), Grenoble, France; ^2^CIC-INSERM 1402, Service de Neurologie, CHU de Poitiers, Université de Poitiers, Poitiers, France; ^3^Department of Psychology, Faculty of Biology, University of Cambridge, Cambridge, United Kingdom

**Keywords:** Parkinson’s disease, impulse control disorders, impulsive choice, delay discounting task, dopaminergic nigrostriatal system, 6-OHDA, rats

## Abstract

Impulse control disorders (ICDs) are frequent behavioral complications of dopaminergic (DA) replacement therapies (DRTs) in Parkinson’s disease (PD). Impulsive choice, which refers to an inability to tolerate delays to reinforcement, has been identified as a core pathophysiological process of ICDs. Although impulsive choices are exacerbated in PD patients with ICDs under DRTs, some clinical and preclinical studies suggest that the DA denervation of the dorsal striatum induced by the neurodegenerative process as well as a pre-existing high impulsivity trait, may both contribute to the emergence of ICDs in PD. We therefore investigated in a preclinical model in rats, specifically designed to study PD-related non-motor symptoms, the effect of nigrostriatal DA denervation on impulsive choice, in relation to pre-existing levels of impulsivity, measured in a Delay Discounting Task (DDT). In this procedure, rats had the choice between responding for a small sucrose reinforcer delivered immediately, or a larger sucrose reinforcer, delivered after a 0, 5, 10 or 15 s delay. In two different versions of the task, the preference for the large reinforcer decreased as the delay increased. However, and in contrast to our initial hypothesis, this discounting effect was neither exacerbated by, or related to, the extent of the substantia nigra pars compacta (SNc) DA lesion, nor it was influenced by pre-existing variability in impulsive choice. These results therefore question the potential implication of the nigrostriatal DA system in impulsive choice, as well as the DA neurodegenerative process as a factor contributing significantly to the development of ICDs in PD.

## Introduction

Parkinson’s disease (PD) is a neurodegenerative disorder hitherto considered to stem from the loss of dopaminergic (DA) neurons in the substantia nigra pars compacta (SNc) and mainly characterized by cardinal motor symptoms (Samii et al., 2004). However, PD is also associated with a plethora of neuropsychiatric deficits, ranging from apathy and depression to impulse control disorders (ICDs; Voon et al., 2011; Sinha et al., 2013; Sierra et al., 2015; Houeto et al., 2016). ICDs are a complex group of impulsive/compulsive behaviors that includes gambling disorder, hypersexuality or compulsive shopping. ICDs are displayed by up to 14%–15% of PD patients under DA replacement therapies (DRTs) of whom quality of life they dramatically affect (Voon et al., 2009; Weintraub et al., 2010; Houeto et al., 2016). At a neurobiological level, despite the suggestion that an overstimulation of the mesocorticolimbic DA system by DRTs promotes ICDs in PD (Dagher and Robbins, 2009; Tang and Strafella, 2012), the underlying psychobiological and etiopathogenic factors that contribute to the development of ICDs only in vulnerable individuals remain unclear.

However, impulsive choice, a form of cognitive impulsivity which reflects an inability to tolerate delays to reinforcement, has been identified as a core pathophysiological process of ICDs (Voon et al., 2011; Houeto et al., 2016). Indeed, impulsive choice as measured in Delay Discounting Tasks (DDTs), which is characterized by the preference for small, immediate, rewards, over larger, delayed, rewards, is exacerbated in PD patients with ICDs (reviewed in Voon et al., 2011). Moreover, higher levels of impulsive choice have also been observed in *de novo* unmedicated or “off” medication PD patients without ICDs compared to healthy controls (Milenkova et al., 2011; Al-Khaled et al., 2015), suggesting that nigral DA cell loss itself may contributes to alter impulse control in PD (Voon and Dalley, 2011).

Recent studies in rats also support this hypothesis. Indeed, bilateral 6-hydroxydopamine (6-OHDA) lesions of the dorsal striatum reduced their tolerance for delayed reinforcers in a DDT (Tedford et al., 2015). In addition, α-synuclein-induced nigrostriatal neurodegeneration has been shown to increase other forms of impulsive behaviors (Engeln et al., 2016). However, the lesional approaches used in these studies provoked substantial motor deficits that may have bias measures of impulsivity. In addition, since only a subset of PD patients is affected by ICDs, the degeneration of the nigrostriatal DA system does not appear to be sufficient to promote ICDs, indicating a potential interaction with an endophenotype of vulnerability, as suggested in a previous study (Engeln et al., 2016).

Because impulsivity is tightly associated with ICDs and represents an endophenotype of vulnerability to develop compulsive behaviors and is a critical factor for the development of compulsive behaviors (Belin et al., 2008; Ansquer et al., 2014), it has been hypothesized that a high impulsivity trait may be associated with the disease progression and the emergence of ICDs in those vulnerable PD patients (Dagher and Robbins, 2009; Voon and Dalley, 2011; Houeto et al., 2016). Yet, the potential relation between the DA denervation and impulsivity remains to be established.

**Figure 1:**
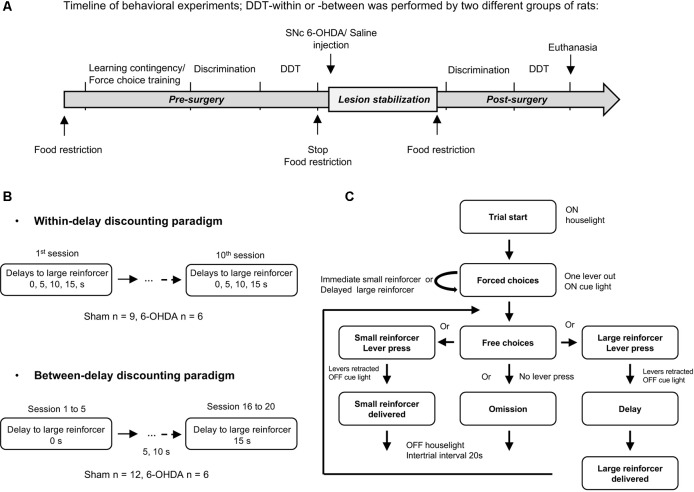
Delay Discounting Task (DDT) paradigms. **(A)** Experimental schedule and timeline of the experiments. Training in DDTs was performed prior to, and after, intra-substantia nigra pars compacta (SNc) 6-hydroxydopamine (6-OHDA)/saline injection. The two experimental groups of rats followed the same timeline at the exception that they were tested either in a DDT-within or DDT-between procedure. **(B)** Flow chart of delay discounting blocks. Each block starts with forced choices wherein only one lever is extended at the time in a random order, allowing rats to learn or recall the contingency of that lever (small or large reinforcer; i.e., 120 μL of a 5% or 10% sucrose solution, respectively) and the delay associated with. Then, rats have free choices access to the small or large reinforcer (both levers are extended), associated with the delay experienced during forced choice trials. No lever press during 35 s of light on period leads to an omission and no reinforcer is delivered. After 35 s, the houselight is turned off and levers are retracted, for a 20 s intertrial interval (ITI), until the beginning of a new trial. During the task, each lever press leads also to levers retraction. **(C)** Schematic representation of the two DDT paradigms. The within-session delay discounting paradigm (within-DDT), is composed of five blocks, each composed of four forced choices (2 per lever) and 10 free choices. The delay increased progressively from one block to another. The task ends after 70 trials. In the between-session delay discounting paradigm (between-DDT), rats first performed 10 forced choices and then 30 free choices, experiencing only one delay per session. The task ends after 40 trials. These two experiments were independent, and two groups of rats were used, one per paradigm.

We therefore investigated in a longitudinal study (Figure 1A) the effect of nigrostriatal DA denervation on impulsive choice and its relation with endogenous level of impulsivity in DDTs. For this, we used a preclinical model in rats specifically designed to study PD-related non-motor symptoms (Magnard et al., 2016). Based on 6-OHDA-induced bilateral but partial lesions of the nigrostriatal DA system, this model has been demonstrated to reveal denervation-induced behavioral impairments, such as motivational- and affective-related deficits, without displaying significant impairments of motor functions (Carnicella et al., 2014; Drui et al., 2014; Favier et al., 2014).

## Materials and Methods

### Animals

Experiments were performed on male Sprague-Dawley rats (Janvier, France) 8 weeks old (weighting 300 g) at the beginning of the experiment. Twenty-one and 31 rats were used in DDT-within and -between experiments, respectively. They were individually housed under standard laboratory condition (12 h/light/dark cycle, with lights ON at 7 a.m.). They were food restricted at 90% of their free feeding weight during the DDT procedure, but had *ad libitum* access to water. Protocols complied with the European Union 2010 Animal Welfare Act and the new French directive 2010/63, and were approved by the French national ethics committee n° 004.

### Bilateral 6-OHDA Lesion

This procedure has been described extensively elsewhere (Carnicella et al., 2014; Drui et al., 2014; Favier et al., 2017). Briefly, food restriction was suspended 2 days before the beginning of the surgery. Rats were administered with desipramine hydrochloride (15 mg.kg^−1^ subcutaneously; Sigma-Aldrich, St. Quentin-Fallavier, France) 30 min before they received an intracerebral infusion of 6-OHDA or vehicle (NaCl) in order to protect noradrenergic neurons. Rats were then anesthetized with a mixture of xylazine (15 mg.kg^−1^) and ketamine (100 mg.kg^−1^) both administered intraperitoneally. Rats were secured on a Kopf stereotaxic apparatus (Phymep, Paris, France) and 6 μg 6-OHDA (Sigma-Aldrich, St. Quentin-Fallavier, France) dissolved in 2.3 μl sterile 0.9% NaCl, or 2.3 μl sterile 0.9% NaCl (sham conditions), were injected bilaterally, through a 26 gauge cannula (Plastic One, Roanoke, USA) in the SNc, at a flow rate of 0.5 μl.min^−1^, at the following coordinates relative to bregma: AP, −5.4 mm; ML, ±1.8 mm; DV, −8.1 mm (Paxinos and Watson, 1998). After recovery from anesthesia, animals were returned to the facilities with food and water available *ad libitum* during 4 weeks in order to allow recovery and the 6-OHDA lesion to develop and stabilize prior to being re-subjected to food restriction and behavioral training.

### Tyrosine Hydroxylase Immunohistochemistry and Quantification of Striatal DA Denervation

Rats were euthanized under chloral hydrate anesthesia at the end of the behavioral experiments. They were intracardially perfused with paraformaldehyde (PFA; 4%) as in Drui et al. (2014; DDT-within) or brains were post-fixed with PFA as in Favier et al. (2017, DDT-between), then frozen in cooled isopentane (−40°C) and stored at −30°C. Fourteen micrometer thick serial frontal sections of the striatum were processed with a cryostat (Microm HM 500, Microm, Francheville, France), collected on microscopic slides and stored at −30°C.

Immunostaining was carried out as previously described (Favier et al., 2017). Sections collected on microscope slides were first air-dried and post-fixed with 4% PFA for 10 min, and then washed in PBS. Brain sections were subsequently incubated with an anti-tyrosine hydroxylase (anti-TH) antibody (mouse monoclonal MAB5280, Millipore, France, 1:2,500) and then with a biotinylated goat anti-mouse IgG antibody (BA-9200, Vector Laboratories, Burlingame, CA, USA; 1:500). Immunoreactivity was visualized with avidin-peroxidase conjugate (Vectastain ABC Elite, Vector Laboratories Burlingame, CA, USA).

TH immunoreactivity in the dorsal striatum and the nucleus accumbens (NAcc) was analyzed across the sections ranging from +2.2 to +0.7 mm from bregma with the ICS FrameWork computerized image analysis system (Calopix, 2.9.2 version, TRIBVN, Châtillon, France) coupled to a light microscope (Nikon, Eclipse 80i) and a Pike F-421C camera (ALLIED Vision Technologies, Stadtroda, Germany) for digitalization. Masks from the different striatal subregions were drawn with the computer analysis system to ensure that appropriate comparisons were made between homologous anatomical regions. Optical densities (ODs) were measured for each striatal region, and the mean OD was calculated with ICS FrameWork software (TRIBVN, 2.9.2 version, Châtillon, France). ODs were expressed as percentages relative to the mean optical density values obtained from the homologous regions of sham-operated animals. Only individuals displaying mean bilateral TH immunoreactivity (TH-IR) loss in the range of 50% to 85% in the dorsal striatum and less than 30% in the NAcc, were included in the analysis, as previously described (Drui et al., 2014; Favier et al., 2014). Based on these restrictive criteria, six and 13 6-OHDA lesioned rats were excluded for the within-DDT and the between-DDT experiments, respectively.

### Delay Discounting Tasks

Sixteen operant chambers for rats (30 × 24 × 27 cm) from Med-Associates (St. Albans, VT, USA) were used. They were equipped with two retractable levers, a cue light located above each lever, a central house light, as well as a dual cup liquid receptacle (ENV-200R3AM) located between the two levers and connected through tubing (PHM-122-18) to syringe pumps (PHM-100) for the delivery of the sucrose solution (5% or 10% w/v in tap water, Sigma-Aldrich, St. Louis, MO, USA).

Two independent delay discounting procedures were carried out on two different batches of rats: one with the delay increasing *within* session (within-DDT) and the other with the delay increasing *between* sessions (one delay at a time, between-DDT; Figure 1B). The two tasks are described below, and were adapted from Evenden and Ryan (1996) and Mar and Robbins (2007) and based on pilot parametric experiments. The procedures started after 1 week of food restriction. Rats were randomly assigned to one operant chamber and the lever side (left or right) assigned to the large or small reinforcer was counterbalanced across operant chambers to prevent any bias of preference. Rats were exposed to one training session each day.

#### Phase 1: Operant and Forced Choice Training

Each session of this phase was divided in 70 and 40 trials for the within-DDT and between-DDT experiment, respectively. When a trial started, *only one* lever was extended and the above cue-light as well as the house light were illuminated to signal the opportunity to press. If the rat pressed the extended lever within 35 s, the lever was retracted, the cue-light turned off and the small reinforcer (120 μL of a 5% sucrose solution) delivered. After 35 s, the house light was turned off for a 20 s intertrial interval (ITI). If the rat failed to press the extended lever during the allocated 35 s period, the lever was retracted, the cue-light turned off at the beginning of the ITI, and an omission was counted.

As soon as the contingency between the instrumental response and the delivery of the small reinforcer (>85% of reinforced trials over three consecutive days) was acquired, rats were trained to acquire the other contingency whereby under similar forced choice sessions, they were required to respond on the other lever to obtain the larger reinforcer (120 μL of a 10% sucrose solution), until the same criteria was reached.

#### Phase 2: Discrimination and Free Choice Training

During this phase, rats were trained to choose between the large and the small reinforcer-associated lever, without any delay. Discrimination phase was considered as acquired when preference for the large reinforcer was above 85% during three consecutive days. As for phase 1, the trial started with the illumination of the house light (35 s long) and it is followed by a 20 s-off period signaled by the absence of light. The within- and between-session procedures are detailed below.

#### Within-Session

The task was divided into five blocks of 14 trials (total: 70 trials/session). Each block began with four forced choices (2 per lever) assigned in a random order, in which only one lever was extended at a time. The remaining 10 trials offered free choices during which both levers and cue-lights were extended and illuminated, respectively. Upon pressing one lever, both levers were retracted immediately and the associated reinforcer (120 μL of a 5% or a 10% sucrose solution) delivered. Each block had the same structure.

#### Between-Sessions

The task started with 10 forced choices (five per lever) assigned in a random order, in which only one lever was extended at the time. Then, rats had access to 30 free choices trials (total: 40 trials/session) with both levers extended and cue-lights illuminated as for the within-session protocol. As in this procedure, only one delay per session was experienced (see “Phase 3: Delay Discounting” section), the last 3 days of the discrimination phase were used to determine the preference for the large reinforcer at the 0 s delay.

#### Phase 3: Delay Discounting

The same blocks and structures as in the discrimination phase were used, except that delays between lever press and the delivery of the large reinforcer varied (Figure 1C).

#### Within-Session (Figure 1B Top)

Rats underwent 10 consecutive delay discounting test sessions, in which delay between lever press and the delivery of the large reinforcer increased between each block in an ascending order (see Tedford et al., 2015 for rationale) within the session. The delays per block were set at 0, 5, 10 and 15 s respectively. The last five sessions were averaged to determine the preference for the large reinforcer in function of delay, as performance was stable across the population (<20% variability between the mean of each group across these five sessions).

#### Between-Sessions (Figure 1B Bottom)

The delay preceding the delivery of the large reinforcer increased every five sessions, with the delays being the same as those used in for the within-session procedure. The last three sessions for each delay were averaged to determine the preference for the large reinforcer, as performances were stable (<20% variability of the mean of each group across these three sessions).

The two DDTs were first performed before the lesion procedure in order to determine individual impulsivity traits and equilibrate the different experimental groups (ANOVAs conducted on this pre-surgery period revealed a main effect of delay: *F*s > 10.56, *p*s < 0.001, but no group effect: *F*s < 0.29, *p*s > 0.59 and no group × delay interaction: *F*s < 0.14, *p*s > 0.93). Post-surgery resumption started on phase 2 (discrimination) in order to evaluate the effect of the lesion on impulsive choice and impulsivity trait.

### Data and Statistical Analyses

Data are presented as mean ±SEM or individual datapoints. Calculation of the area under the curves (AUC) in order to measure delay discounting in each individual, were adapted from Myerson et al., 2001. Briefly, delays, and large reinforcer preference were first expressed as the proportion of their maximum value in order to be comprised between 0 and 1. Then, the resulting discounting curve was subdivided discounting graph into a series of half trapezoids, from 0 to 5 s, 5 to 10 s and 10 to 15 s. The area of each trapezoid is thus equal to the following equation: x_2_ − x_1_(y_1_ + y_2_)/2, where x_1_ and x_2_ are successive delays associated with the large reinforcer preference y_1_ and y_2,_ respectively. Thus, the area under the discounting curve is the sum of the area of the three trapezoids. In contrast to Myerson et al. (2001), the value of the delay 0 was not normalized to 100% (i.e., 1), because the current strategy is considered better to capture the reaction and adaptation of each individual to the delay with regards to their initial preference for the large reinforcer. Although we found a strong correlation between the two measures of AUC (*R*^2^ = 0.88 and 0.82 for the within-session and between-sessions DDTs experiments respectively), these two calculation methods may lead to slight differences. Hence, measures of DD-AUC using the normalization described by Myerson et al. (2001), are reported in Supplementary Figure S2.

Data were analyzed by *t*-test or two-way repeated measure ANOVAs, using SigmaStat software (SigmaStat 4.0 2016, Systat software Inc., San Jose, USA). When indicated, *post hoc* analyses were carried out using the Student-Newman-Keuls test. Correlations were performed and analyzed with parametric linear regression approaches (Pearson product moment correlation and comparison of linear regression coefficients). Assumptions for the normality of the distributions and the homogeneity of variance were verified using the Shapiro-Wilk and Levene test, respectively. Significance for *p* values was set at *α* = 0.05. Effect sizes for the ANOVAs are also reported using partial *η*^2^ values (Levine and Hullett, 2002; Murray et al., 2015).

## Results

**Figure 2:**
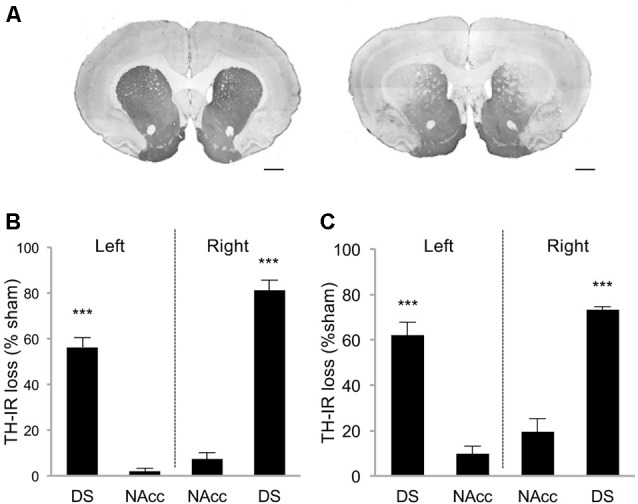
Striatal tyrosine hydroxylase immunoreactivity (TH-IR) loss induced by 6-OHDA SNc lesion. **(A)** Representative photomicrographs of TH-immunostained striatal coronal section of sham rat (left picture) and 6-OHDA lesioned rat (right picture). Quantification of TH-IR loss in DDT within-session experiment **(B)** and between-session experiment **(C)**. Data are expressed as the percentage of the mean optical density value obtained for sham rats on the AP levels +2, 2; +1, 6; +0, 7. ±SEM. ***p < 0.001. DS, Dorsal striatum; NAcc, Nucleus accumbens. Scale bar: 1 mm.

Rats were first trained in a DDT under which the delay increased progressively within each test session (within-DDT), in order to measure individuals’ basal level of impulsive choice and distribute them in the various experimental groups. Rats were subsequently exposed to either sham or bilateral infusions of 6-OHDA into the SNc, which resulted in partial selective DA denervation in the dorsal striatum that relatively spared the NAcc (68.8 ± 2.4% and 4.6 ± 1.8% loss of TH-immunolabeling, respectively, (*t*_(10)_ = 21.42, *p* < 0.001; Figures 2A,B). This pattern of denervation is in line with previous studies (Drui et al., 2014; Favier et al., 2014) which have demonstrated it circumvents the motor deficits frequently associated with greater or other patterns of nigrostriatal DA lesions.

**Figure 3:**
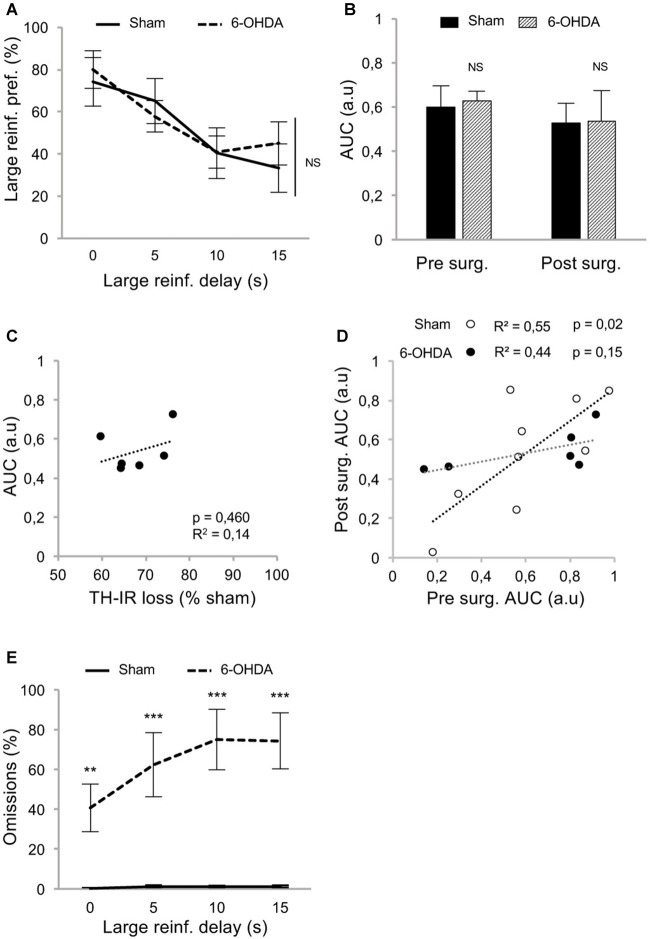
Partial nigrostriatal denervation does not affect impulsivity in a within-session DDT (within-DDT). **(A)** Similar discounting pattern between sham and 6-OHDA lesioned rats, expressed as percentage of preference for the large reinforcer over the smaller one, in function of delay. In this procedure, rats experienced all delays in each session. Data are represented as mean ±SEM and were averaged from the last five sessions. **(B)** Similar AUC, between sham and 6-OHDA lesioned rats, pre- and post-surgery period. The AUC is extrapolated from area under discounting curves and expressed as mean value ±SEM for each group. **(C)** No correlation between the post-surgical AUC and the degree of dorsal striatum TH-IR loss. Dots represent individual values for the AUC and dorsal striatum TH-IR loss expressed as percentage of sham mean value. **(D)** Positive correlation between pre and post-surgery of individual AUC values for sham operated rats (empty circle) and 6-OHDA lesioned rats (full circle). Correlation is only significant for sham rats (*p* < 0.05; for 6-OHDA rats: *p* = 0.15). **(E)** 6-OHDA lesioned rats exhibited a higher percentage of omissions than sham rats during within-DDT. Data are represented as mean of omissions ±SEM in function of delay (averaged for the last five sessions). NS, non-significant, **p < 0.01; ***p < 0.001; Sham (*n* = 9) vs. 6-OHDA (*n* = 6), reinf, reinforcer; AUC, area under the discounting curve; a.u, arbitrary units.

At resumption of within-DDT training, all rats maintained a preference for the large reinforcer over the smaller one in absence of delay, and irrespective of the lesion (Figure 3A; delay 0 s), indicating that, in agreement with previous results (Drui et al., 2014), the relative reinforcing value of natural rewards was not influenced by the nigrostriatal DA denervation. This preference for the large reinforcer decreased as the delay increased (main effect of delay: *F*_(3,39)_ = 9.94, *p* < 0.001, partial *η*^2^ = 0.43; Figure 3A). However, this discounting effect was not exacerbated in lesioned animals (no effect of lesion: *F*_(1,39)_ = 0.05, *p* = 0.83, partial *η*^2^ = 0.006, and no delay × lesion interaction: *F*_(3,39)_ = 0.49, *p* = 0.69, partial *η*^2^ = 0.04; Figure 3A), which led eventually to a similar AUC in both groups (no effect of lesion: *F*_(1,26)_ = 0.03, *p* = 0.86, partial *η*^2^ = 0.001, and no period × lesion interaction: *F*_(1,26)_ = 0.01, *p* = 0.92, partial *η*^2^ < 0.001; Figure 3B; see also Supplementary Figure S2A). Level of impulsivity seemed not to be related to the extent of the nigrostriatal DA denervation, as no correlation was evidenced between the percentage of TH-immunolabeling loss in the dorsal striatum and the level of impulsive choice (indexed by the AUC; Figure 3C and Supplementary Figure S2B). A positive correlation was nevertheless found between the AUC before and after surgery in sham rats (Figure 3D). Intriguingly, this relation significantly decreased in the 6-OHDA condition (significant difference in regression slope: *t*_(11)_ = 3.35, *p* < 0.05; Figure 3D), which may indicate a narrowing of the variance of this trait or a change in the strategy used to compute the delay-preference function. However, this relation as well as the differences between sham vs. lesion groups, were not found with the conventional normalization method of Myerson et al. (2001; Supplementary Figure S2C).

In addition, although the number of omissions displayed by the sham group remained very low in both forced- and free-choice trials and across delays (Supplementary Figures S1A,B, Figure 3E, respectively), it was considerably increased in lesioned rats, especially as the session progressed and the delay increased (for free-choice trials, main effect of the lesion: *F*_(1,39)_ = 31.83, *p* < 0.001, partial *η*^2^ = 0.91 and delay × lesion interaction: *F*_(3,39)_ = 15.75, *p* < 0.001, partial *η*^2^ = 0.55). This increase in omissions may reflect an increased aversion to the cognitive/motivational demand when delays are introduced, which results in a delay-dependent disengagement from the task or a more general impairment in maintaining a motivated behavior over prolonged periods of time. Interestingly, a systematicity in the omissions profile was observed, as even within a block, omissions mostly occur at the end. Indeed, such SNc DA lesions have been shown to impaired the maintenance of preparatory and seeking behaviors (Magnard et al., 2016; Favier et al., 2017) and nigrostriatal DA denervation can induce profound attentional and/or cognitive deficits (Nieoullon and Coquerel, 2003; Aarts et al., 2011).

**Figure 4:**
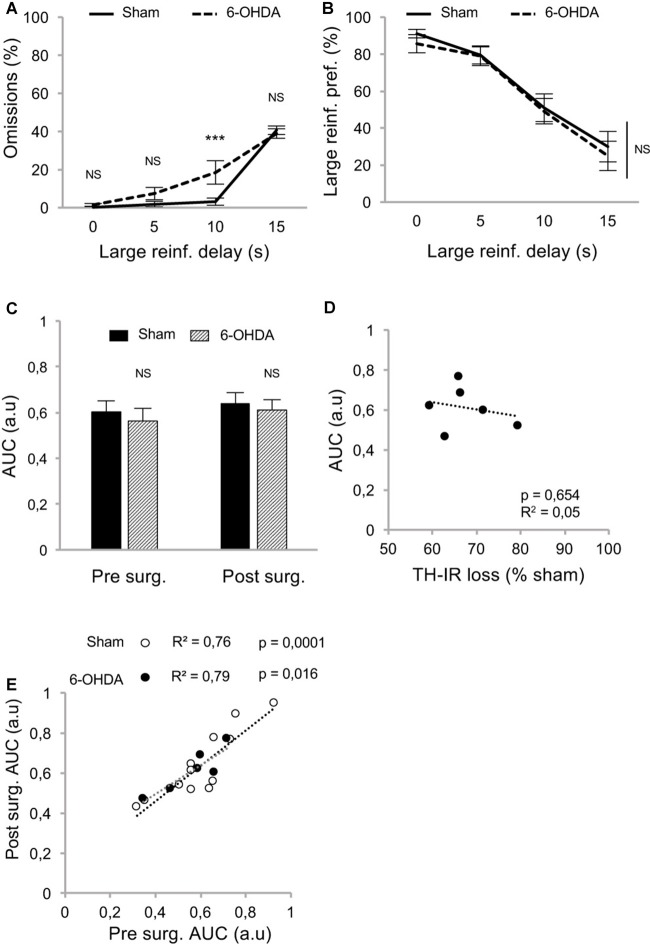
Partial nigrostriatal denervation does not affect impulsivity in a between-session DDT (between-DDT). **(A)** Percentage of omissions in sham and 6-OHDA lesioned rats during between-DDT. Data are represented as mean of omissions ±SEM in function of delay (averaged from each delay from the last three sessions). **(B)** Similar discounting pattern between sham and 6-OHDA lesioned rats, expressed as percentage of preference for the large reinforcer over the smaller one, in function of delay. Data are represented as mean ±SEM and were averaged from the last three sessions for each delay. **(C)** Similar discounting AUC, between sham and 6-OHDA lesioned rats, pre- and post-surgery period. The AUC is extrapolated from AUC and expressed as mean value ±SEM for each group. **(D)** No correlation between the post-surgical AUC and the degree of dorsal striatum TH-IR loss. Dots represent individual values for the AUC and dorsal striatum TH-IR loss expressed as percentage of sham mean value. **(E)** Positive and significant correlation between pre and post-surgery of individual AUC values for sham and 6-OHDA lesioned rats (*p*s < 0.05). NS, non-significant, ***p < 0.001; Sham (*n* = 12) vs. 6-OHDA (*n* = 6), reinf, reinforcer; AUC, area under the discounting curve; a.u, arbitrary units.

Because such a high level of omissions may have interfered with the discounting data by biasing the sampling of the choice responses by the rats, we modified the task to limit this effect. We hypothesized that testing only one delay at a time by increasing delays across sessions (between-DDT), would make each session shorter and less taxing. This new procedure was tested with another batch of rats with a similar pattern of DA denervation (Figure 2C). Even if lesioned rats displayed significantly higher levels of omissions than those of the sham group during forced- (Supplementary Figures S1C,D) and free-choice trials (main effect of lesion: *F*_(1,48)_ = 5.62, *p* < 0.05, partial *η*^2^ = 0.18, of the delay: *F*_(3,48)_ = 126.49, *p* < 0.001, partial *η*^2^ = 0.89, and delay × lesion interaction: *F*_(3,48)_ = 5.64, *p* < 0.05, partial *η*^2^ = 0.26; Figure 4A), this effect was markedly reduced, as the difference between the groups was attributable only to performance on the 10 s delay. Even under these conditions, which controlled for the potential confounding influence of high omissions in lesioned rats, these data confirmed that the partial bilateral SNc DA lesion did not exacerbate impulsive choice (main effect of delay: *F*_(3,48)_ = 54.12, *p* < 0.001, partial *η*^2^ = 0.77, but no effect of lesion: *F*_(1,48)_ = 0.20, *p* = 0.66, partial *η*^2^ = 0.01, or delay × lesion interaction: *F*_(3,48)_ = 0.09, *p* = 0.96, partial *η*^2^ = 0.006; Figure 4B). This was further supported by an absence of difference between sham and lesioned rats in the AUC (no effect of lesion: *F*_(1,32)_ = 0.35, *p* = 0.55, partial *η*^2^ = 0.01, and no period × lesion interaction: *F*_(1,32)_ = 0.19 *p* = 0.66, partial *η*^2^ = 0.001; Figure 4C; see also Supplementary Figure S2D) and, at the population level, by the absence of relationship between DA denervation and AUC (Figure 4D and Supplementary Figure S2E).

In addition, and in contrast with the previous experiment, the lesion did not change the correlation between pre- and post-surgery AUC, indicating no influence on impulsivity trait in individuals (no difference in regression slope: *t*_(14)_ = 0.78, *p* > 0.40; Figure 4E; see also Supplementary Figure S2F).

## Discussion

Using a validated 6-OHDA lesion-based rodent model that was specifically designed for the investigation of non-motor, neuropsychiatric impairments related to PD (reviewed in Cenci et al., 2015; Magnard et al., 2016), we showed that bilateral and partial denervation of the nigrostriatal DA pathway neither induced nor exacerbate impulsive choice in two different DDTs, taking into account inter-individual variability at baseline.

The AUC, was used here as an empirical objective measure of discounting behavior and impulsivity trait in rats (Myerson et al., 2001; Odum, 2011). The discounting rate k factor has hitherto been a preferred index in clinical studies to assess intertemporal choice (e.g., Milenkova et al., 2011; Al-Khaled et al., 2015). Here, AUC was preferred to the k factor as an index of impulsivity, because it better accommodates the properties of the current dataset. Indeed, calculation of the k factor derives from the slope of the discounting function and relies on the indifference point (Broos et al., 2012), which is not necessarily crossed by all rats (especially low impulsive animals) under our experimental conditions. Together with the marked inter-individual differences observed in the present study in the time course of the discounting curve a fit-for-all model could not be implemented here. Nevertheless, the strong correlations observed between AUC obtained at different time points and test phases in sham conditions, especially in the between-sessions DDT, offer further evidence that it is a relevant and reliable measure of impulsive choice for longitudinal studies, as well as a useful and alternative tool for identifying endophenotypes of impulsive choice, in rats.

Although the discounting of the larger reinforcer over increasing delays was not influenced by nigrostriatal DA denervation, its inter-individual variability was markedly decreased by the lesion in the within- but not between-DDT. Interestingly, bilateral excitotoxic lesions of the dorsal striatum have also been shown to discretely flatten delay-preference function without affecting delay discounting in a within-session design version of the task (Dunnett et al., 2012). Because this effect progressively disappeared with extensive training, it had been attributed to a decrease ability to adjust behavior to the rapid modifications of task parameters across test sessions rather than to an alteration of impulsivity *per se* (Dunnett et al., 2012). Using similar experimental conditions (e.g., food restriction, similar pattern of DA denervation), the number of omissions drastically decreased in a between-DDT, in which the behavioral/motivational (less trials and shorter sessions) and cognitive (only one delay tested at any given time) demand, was reduced compared to a within-DDT. Consistently, Tedford et al. (2015) did not report any significant increase in omissions in another between-sessions DDT following dorsostriatal DA lesions. Together, these data suggest that nigrostriatal DA denervation may decrease the ability of the animal to properly engage in the task, and that between-session designs may be more appropriate than within-session designs to investigate the contribution of the nigrostriatal DA system to impulsive choice.

Although some clinical studies suggest a possible role of the neurodegenerative process in the development of impulsive behaviors in PD (Milenkova et al., 2011; Al-Khaled et al., 2015), conflicting results have been reported about the potential implication of a nigrostriatal DA deficit in different forms of impulsivity (Rokosik and Napier, 2012; Tedford et al., 2015; Engeln et al., 2016; Carvalho et al., 2017). This likely stems from the difficulty to disentangle an effect of nigrostriatal DA denervation on impulse control from its often dramatic effect on motor performance, alongside with the cognitive or motivational alterations potentially induced by DA lesions (Cenci et al., 2015).

Notably, it has been observed that a bilateral DA lesion of the dorsal striatum increased delay discounting in a between-session, wherein a similar range of ascending delays as the one used in the present study was applied (Tedford et al., 2015). However, substantial methodological differences between the two studies may account for these seemingly contradictory results. First, the retrograde lesion approach used in Tedford’s study led to motor dysfunctions, which could have impacted the coordinated sequences of actions necessary to perform the associated chained scheduled task. In addition, the use of a dorsostriatal retrograde lesion as opposed to the SNc anterograde lesion performed in the present study may lead to a different DA denervation pattern (e.g., Cenci et al., 2015) or different underlying DA deficits and compensatory mechanisms. Tedford et al. (2015) also used intracranial self-stimulation (ICSS) as a reinforcer in order to avoid satiety and other potential issues associated with food. However, chronic ICSS itself can induce profound neuroadaptations, such as overexpression of DA D_1_ receptors in the NAcc (Simon et al., 2016), a structure in which DA receptors modulate impulsive choice (Basar et al., 2010). Therefore, the increased delay discounting reported in this study may result from a direct neurobiological interaction between ICSS and the lesion.

Although the present study focuses exclusively on nigrostriatal cell loss and the emergence of impulsive choice, it should be kept in mind that other neurochemical systems, such as the serotoninergic and noradrenergic systems, have been shown to be affected by the neurodegenerative processes of PD (Delaville et al., 2011; Maillet et al., 2016). Due to their implication in the control of impulsive behaviors, alteration of these two monoaminergic systems, which, in the case of noradrenaline, may even precede the degeneration of DA neurons (Delaville et al., 2011), are prone to contribute, independently, or in conjunction with DA denervation, to the development of impulsivity in PD (Dalley and Roiser, 2012; Kehagia et al., 2014; Ye et al., 2014; Dalley and Robbins, 2017).

Nevertheless, our study provides novel insights into the contribution of the nigrostriatal DA system to impulsive choice and useful methodological considerations for future studies. It also highlights the fact that further investigations are necessary to better apprehend the potential contribution of the DA neurodegenerative process in conjunction with impulsivity trait and DRTs to the development of ICDs in PD.

## Author Contributions

RM, J-LH, MS, DB and SC designed research. RM, YV, CC, SB and SC performed research. RM, CC and SC analyzed data. RM, DB and SC wrote the manuscript with the help of the other authors.

## Conflict of Interest Statement

The authors declare that the research was conducted in the absence of any commercial or financial relationships that could be construed as a potential conflict of interest.
