# Supplementary material for: Nigrostriatal Dopaminergic Denervation Does Not Promote Impulsive Choice in the Rat: Implication for Impulse Control Disorders in Parkinson’s Disease
Source: Front Behav Neurosci. 2018 Dec 13;12:312. doi: 10.3389/fnbeh.2018.00312 (PMC6300586; doi:10.3389/fnbeh.2018.00312)
Supplement: Supplementary file 1 [file Data_Sheet_1.docx]

**Supplemental figure legend**

**Figure S1. Omissions during forced choice trials.** Percentage of omissions in sham and 6-OHDA lesioned rats during within-DDT for the small (**A**) and large (**B**) reinforcer associated lever. Data are represented as mean of omissions $\text{±}\text{ SEM}$ in function of delay (averaged from each delay from the last 5 sessions). Percentage of omissions in sham and 6-OHDA lesioned rats during between-DDT for the small (**C**) and large (**D**) reinforcer associated lever. Data are represented as mean of omissions $\text{±}\text{ SEM}$ in function of delay (averaged from each delay from the last 3 sessions). N.S: non-significant; **p<0,01; ***p<0.001. Reinf.: reinforcer.

**Figure S2. Delay-discounting (DD)-related AUC quantification with alternative normalization method (Myerson et al., 2001) for the within- (A-C) and between-DDT (D-F).** (**A**) Similar AUC, expressed as mean value $\text{±}\text{ }\text{SEM}$ [no effect of lesion: F(1, 26) = 0.01, p = 0.99, partial η^2^ < 0.001, and no period x lesion interaction: F(1, 26) = 0.04 p = 0.83, partial η^2^ = 0.003]. (**B**) No correlation was observed between the post-surgical AUC and the extent of tyrosine hydroxylase immunoreactivity (TH-IR) loss in the dorsal striatum. Dots represent individual values for the AUC and dorsal striatum TH-IR loss expressed as percentage of sham mean value. (**C**) No correlation was observed between pre and post-surgery individual AUC values for sham operated rats (empty circle) and 6-OHDA lesioned rats (full circle). (**D**) Similar AUC, expressed as mean value $\text{±}\text{ SEM}$, was observed between sham and 6-OHDA lesioned rats across pre- and post-surgery periods [no effect of lesion: F(1, 32) = 0.01, p = 0.93, partial η^2^ = 0.003, and no period x lesion interaction: F(1, 32) = 0.71 p = 0.41, partial η^2^ = 0.04]. (**E**) No correlation was observed between the post-surgical AUC and the extent of TH-IR loss in the dorsal striatum. Dots represent individual values for the AUC and dorsal striatum TH-IR loss expressed as percentage of sham mean value. (**F**) A positive correlation was observed between pre and post-surgery of individual AUC values for sham operated (empty circle) and 6-OHDA lesioned rats (full circle). NS, non-significant; Within-DDT: Sham (n = 9) vs. 6-OHDA (n=6) and Between-DDT: Sham (n = 12) vs. 6-OHDA (n=6), reinf: reinforcer, AUC: area under the discounting curve, a.u: arbitrary units.
