# Supplementary figures and images for: Nigrostriatal Dopaminergic Denervation Does Not Promote Impulsive Choice in the Rat: Implication for Impulse Control Disorders in Parkinson’s Disease
Source: Front Behav Neurosci. 2018 Dec 13;12:312. doi: 10.3389/fnbeh.2018.00312 (PMC6300586; doi:10.3389/fnbeh.2018.00312)

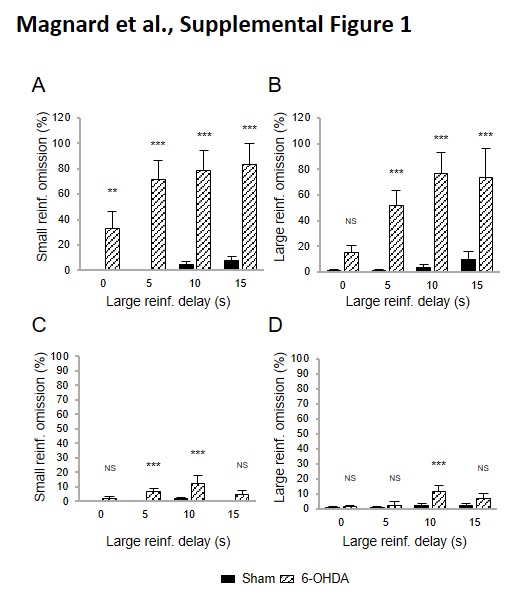

Supplement: Supplementary file 2 [file Image_1.jpeg]

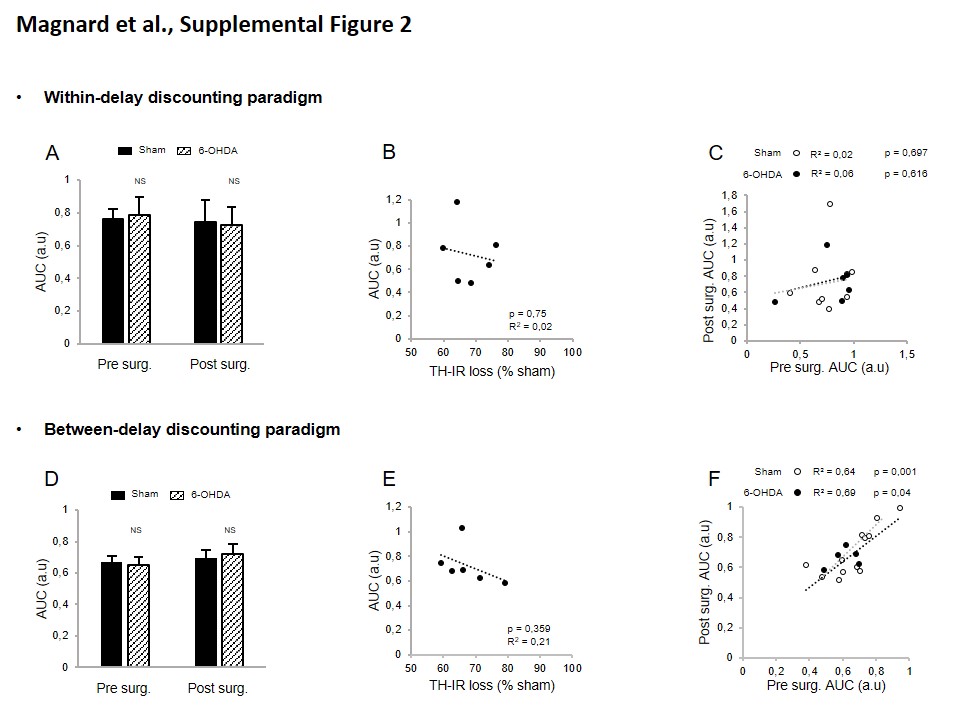

Supplement: Supplementary file 3 [file Image_2.jpeg]
